# Mortality Measurement Matters: Improving Data Collection and Estimation Methods for Child and Adult Mortality

**DOI:** 10.1371/journal.pmed.1000265

**Published:** 2010-04-13

**Authors:** Colin Mathers, Ties Boerma

**Affiliations:** Department of Health Statistics and Informatics, World Health Organization, Geneva, Switzerland

## Abstract

Colin Mathers and Ties Boerma discuss three research articles in *PLoS Medicine* that address the measurement and analysis of child and adult mortality data collected through death registration, censuses, and household surveys.

**Linked Research Articles**

This Perspective discusses the following new studies published in *PLoS Medicine*:

1. Murray CJL, Rajaratnam JK, Marcus J, Laakso T, Lopez AD (2010) What Can We Conclude from Death Registration? Improved Methods for Evaluating Completeness. PLoS Med 7(4): e1000262. doi:10.1371/journal.pmed.1000262
  Murray and colleagues evaluate the performance of a suite of demographic methods which estimate the fraction of deaths registered and counted by civil registration systems, and identify three variants that generally perform the best.
2. Rajaratnam JK, Tran LN, Lopez AD, Murray CJL (2010) Measuring Under-Five Mortality: Validation of New Low-Cost Methods. PLoS Med 7(4): e1000253. doi:10.1371/journal.pmed.1000253
  Using data from 166 Demographic and Health Surveys, Rajaratnam and colleagues develop and validate new empirically based methods of estimating under-five mortality.
3. Obermeyer Z, Rajaratnam JK, Park CH, Gakidou E, Hogan MC, et al. (2010) Measuring Adult Mortality Using Sibling Survival: A New Analytical Method and New Results for 44 Countries, 1974–2006. PLoS Med 7(4): e1000260. doi:10.1371/journal.pmed.1000260
  Obermeyer and colleagues describe a novel method, called the Corrected Sibling Survival method, to measure adult mortality in countries without good vital registration by use of histories taken from surviving siblings.

The accurate measurement and estimation of mortality levels, trends, causes, and differentials are a cornerstone of public health. Child and adult mortality rates, often summarized in a life expectancy measure, are key indicators of levels of health and development. The preferred source of mortality data is prospective measurement through continuous registration of deaths, as is done in civil registration systems. But in many countries, especially those with poorly developed statistical systems and higher levels of mortality, retrospective measurement in households and surveys is the principal vehicle for data collection. All methods of data collection suffer from two generic problems: omission of events and dating errors. During the past few decades, demographers have developed and used a range of methods to improve data collection, assess levels of bias, and correct for such biases [1]–[3]. In three papers published in this issue of *PLoS Medicine* [4]–[6], Murray, Rajaratnam and colleagues revisit these analytical methods and techniques and present improved methods for the analysis of mortality data collected through death registration, censuses, or household surveys.

## Death Registration Data: Evaluating Completeness

During 1995–2004 countries with complete civil registration systems (over 90% coverage of deaths) were a minority and accounted for only 26% of global deaths, with no progress in four decades [7]. At present, about 100 of the 192 WHO Member States report death registration data to the World Health Organization, and not all of these data are reasonably recent [8]. Data for around 60 of these countries are considered to be essentially complete. If the level of completeness of reporting were known and age reporting were fairly accurate, age- and sex-specific mortality patterns could be estimated for many countries.

Death distribution methods are used to assess completeness of reporting. They involve, in one form or another, comparison of the death numbers across age groups with population estimates. Unfortunately, all these methods depend on assumptions (stable age structure of population, zero migration, no misreporting of ages in deaths or population) that are violated in practice and are known from direct experience to produce results that can be quite uncertain or unreliable. The evaluation in this issue by Murray and colleagues [4] of a large set of death distribution methods shows that the completeness estimates tend to have large uncertainty ranges (of the order of ±20% or greater). These results are sobering for those who use these methods to adjust incomplete death registration data to estimate true mortality rates.

Clearly, the way forward for improving our knowledge of the levels and trends of mortality risks in populations is to encourage direct evaluation studies at national and subnational levels that can directly quantify completeness of death registration. One avenue for doing this is to compare mortality levels derived from death registration data with those derived for children or adults using survey methods. For example, the United Nations Interagency Group on Mortality Estimation compares death registration data and survey data on child mortality to directly assess levels of completeness for some countries [9].

Furthermore, international agencies and global health actors must step up efforts to improve the completeness and quality of death registration systems in all countries, as was argued in a series on birth and death registration in *The Lancet* in 2007 [10],[11].

## Child Mortality

In the absence of complete and accurate prospective systems of data collection, the main methods of collection of child death information are based on questions in surveys and censuses about recent deaths in the household, or on complete and summary birth histories. Collection of data on recent deaths (i.e., in the last 12 months) was popular in the sixties but yielded unsatisfactory results due to serious underreporting of deaths. Complete birth histories, in which a mother is asked questions about dates and survival status of all of her children, became popular during the late seventies in the World Fertility Survey and has been implemented in more than 200 national surveys conducted as part of the Demographic and Health Survey program (DHS) since 1985. These surveys are the main source of monitoring trends in neonatal, infant, and child mortality in developing countries. Furthermore, the individual-level data permitted an extensive body of work on the determinants of child mortality. The main problems refer to omission of events and errors in ages and dates.

Summary birth histories are based on the number of children ever born and those still alive, and do not require information on each child. Data collection is less burdensome for the interviewer and respondent than a complete birth history. However, the analysis, based on methods proposed by William Brass almost 50 years ago [12],[13], requires more assumptions to fill the data gaps. In their study published in *PLoS Medicine* this week, Rajaratnam and colleagues [5] develop methods that enhance the ability to pick up more recent mortality trends and to estimate uncertainty. The methods are considerably more complex than the original methods, involving extensive smoothing and using data from other countries to fill gaps.

The quality of the mortality data gathered through the summary birth history remains a key issue, especially if the data are used to produce local mortality estimates. The proposed methods perform well with the selected datasets, which were DHS surveys that included both full and summary birth histories. In such surveys, the data quality of the summary birth history is likely to be higher because it comes from a full birth history, than it would be in a census or survey where there is no full birth history.

In general, full birth histories should continue to be the recommended method of data collection in surveys, if at all possible. Summary birth histories, however, provide useful information of levels and trends, especially if used in censuses allowing district child mortality estimates, but also in national surveys that can only include a few questions on child mortality. It will be important to provide an easy-to-use tool and training for countries that intend to explore the methods, as indicated by the authors.

## Adult Mortality

Compared to child mortality, the measurement of adult mortality levels and trends has been lagging, but the epidemiological and demographic transition, the interest in measurement of maternal mortality, and the emergence of AIDS as a major killer of young adults have generated much greater interest in the subject. In the absence of complete death registration, adult mortality data are collected in surveys and censuses through questions on recent deaths and survival of parents and of siblings. Sibling history modules have now been included in nearly 100 DHS surveys, primarily driven by the demand to measure maternal mortality, and represent a potentially valuable source of information on levels of adult mortality. To date, limited use has been made of sibling survival data collected in household surveys to estimate levels of adult mortality, largely because of concerns of under-reporting [14]–[17].

The under-reporting bias in reported sibling death data relates in part to mortality bias (high-mortality sibships are less likely to be included due to higher chance of death of all siblings) and in part to under-reporting by respondents who may not know sibling status, or may forget to report some siblings or deaths. Estimation of the death toll due to violence and indirect causes in conflict situations adds additional levels of complexity and bias, as data collection is difficult, events may occur in foci, and traditional models of adjustment do not apply [18].

Gakidou and King have proposed methods to adjust for sampling and mortality biases [19], essentially by reweighting the observations to give lower weight to sibships with a higher number of survivors, and through innovative use of the data to estimate the mortality rate for the sibships with no survivors. Their method was applied to data from the Iraq Family Health Survey to estimate excess deaths in the Iraqi population due to violence in 2003–2006, but the authors concluded that the resulting estimates still needed further adjustment for under-reporting [20].

Obermeyer and colleagues [6] in this issue present a method for adjusting for the under-reporting bias in sibling survival data. The method essentially assumes that the degree of under-reporting increases linearly with time between sibling death and survey interview, and estimates the coefficient through a pooled regression involving all available surveys. The under-reporting coefficient is estimated using data from multiple surveys in the same populations which are reporting on the deaths in the same period with different lags to survey date. Their results suggest more plausible estimates of adult mortality after correction for under-reporting.

The two main limitations of the proposed method are the need to make assumptions on the age pattern of sibling deaths due to small sample sizes in most surveys and the assumptions of linear “forgetting” and the “rate of forgetting” being the same across countries. It may be feasible to address these limitations as the pool of sibling survival data increases with new surveys.

This approach offers the potential to considerably expand the evidence available for assessing and monitoring levels of adult mortality in high-mortality countries, using a method that is reasonably easy to implement. However, the survey time required for the sibling survival module is not trivial, particularly for respondents with many siblings, and is an even greater burden if some form of verbal autopsy is also included to obtain cause-of-death information. It should, however, be encouraged to include sibling survival modules in surveys.

## Conclusion

A priority for the improvement of the measurement of mortality in developing countries should be to increase the empirical underpinnings in countries without high-quality death registration data systems. Such improvements should include promotion of prospective measurement through civil registration systems as a mid- to long-term investment; regular demographic and health surveys with full or, if that is not possible summary, birth histories and sibling survival histories; and decennial censuses with the appropriate mortality questions. All methods suffer, to a varying extent, from the basic problems of omission of deaths and dating errors, as well as method-specific biases. Estimation methods are required to assess completeness of reporting and adjust for under-reporting. These three papers present a welcome effort to improve the analysis of imperfect mortality data.
